# Clinical Decision-Making Case: Thyroid Storm

**DOI:** 10.21980/J8.53003

**Published:** 2025-12-31

**Authors:** Stephanie Cohen, Amrita Vempati, Charles Lei, Hillary Moss, Tiffany Moadel, Suzanne Bentley, Stephanie Stapleton, Kelly Roszczynialski

**Affiliations:** 1 University of Central Florida, Department of Emergency Medicine, Orlando; 2 Creighton School of Medicine Phoenix, Department of Emergency Medicine, Phoenix, AZ; 3 Hennepin County Medical Center, Department of Emergency Medicine, Minneapolis, MN; 4 Montefiore Medical Center, Department of Emergency Medicine, Bronx, NY; 5 Zucker School of Medicine at Hofstra/Northwell, Department of Emergency Medicine, Hempstead, NY; 6 NYC Health + Hospitals, Department of Emergency Medicine, Brooklyn, NY; 7 Boston University/Boston Medical Center, Department of Emergency Medicine, Boston, MA; 8 Stanford University, Department of Emergency Medicine, Palo Alto, CA

## Abstract

**Audience:**

This clinical decision-making case is intended for all emergency physicians (EP) in training.

**Introduction:**

Thyroid storm (TS) is a rare but life-threatening endocrine emergency that represents the most severe form of thyrotoxicosis. If not promptly recognized and appropriately managed, TS carries a mortality rate of up to 25%.^1^ However, with timely and aggressive treatment, mortality can be significantly reduced to 1.2–3.6% in the United States.^2^ Due to its rarity and often nonspecific presentation, early diagnosis and intervention in the emergency department are essential to improving patient outcomes. Recognizing its critical nature, the American Board of Emergency Medicine (ABEM) identifies thyroid storm as a core emergency condition that emergency physicians (EPs) must be trained to manage.^3^ Additionally, recent updates to the ABEM certifying examination emphasize the importance of clinical decision-making and the ability to verbalize diagnostic reasoning and management plans. This case has been designed to help learners practice and demonstrate these skills in the context of a high-stakes, time-sensitive clinical scenario involving thyroid storm.

**Educational Objectives:**

By the end of the session, learners will be able to: 1) verbalize key pertinent historical and physical exam findings in a young female patient presenting with altered mental status; 2) formulate a prioritized differential diagnosis based on the history and physical exam; 3) order appropriate diagnostic studies and recognize abnormalities suggesting thyroid storm; 4) describe pathophysiology, management and rationale of sequential pharmacologic therapy in thyroid storm; 5) communicate patient’s medical care and course to family; and 6) review essential disposition actions including consultations and level of care for admission.

**Educational Methods:**

We implemented a certifying exam board format case aligned with the ABEM’s updated certifying exam board examination. The case was co-developed by experts in simulation-based education and emergency medicine and underwent external peer review, which focused on the accuracy of the clinical context, clarity of scenario instructions, and educational value.

**Research Methods:**

The case was iteratively developed and refined through multi-site piloting. Initial design was completed by three faculty with emergency medicine and simulation backgrounds, followed by external review using the Simulation Scenario Evaluation Tool (SSET). Feedback focused on case progression, realism, alignment with objectives, and consistency with assessment standards.

The case was then piloted at two academic training sites and at the Society for Academic Emergency Medicine (SAEM) Annual Meeting. Participants included faculty facilitators and EM residents. Residents and faculty completed a modified usability survey incorporating Likert-scale items (1 = strongly disagree to 5 = strongly agree) and open-ended comments. Data was collected in Qualtrics^®^ and analyzed in Excel^®^. Revisions were made after each iteration to improve clarity, usability, and educational impact.

**Results:**

The simulation scenario evaluation tool (SSET) evaluations were strongly positive. Facilitators (n = 3) consistently rated the case objectives, critical actions, and supporting materials as clear. They agreed or strongly agreed that the case was appropriate for the learner level, that the clinical course adhered to the ABEM format, and that the critical actions supported the stated objectives. Mean ratings ranged from 4 to 5 for ease of use, and facilitators noted that their colleagues would also find the materials accessible. They described the case as well-integrated, expressed confidence in facilitation, and endorsed its utility for ABEM certifying exam preparation.

Resident feedback was similarly supportive. Three learners unanimously agreed that the case provided helpful practice for the ABEM exam and reported that both the written and verbal instructions were clear.

**Discussion:**

The thyroid storm clinical decision-making case proved to be an effective educational tool, meeting its intended objectives and offering meaningful preparation for emergency medicine residents facing the new ABEM certifying examination. Facilitators consistently reported that the case objectives, critical actions, and supporting materials were clear and aligned with the targeted level of learner training. Similarly, both facilitators and residents found the case to be a valuable exercise, providing relevant and realistic practice in the style of the certifying exam.

While early results are encouraging, future challenges and opportunities remain. In particular, the evolving structure and scoring approach of the new ABEM certifying examination introduces areas where additional refinement may be needed. As more information becomes available regarding the exam’s evaluation metrics, this case can be further tailored to enhance alignment and maximize its educational impact. Continued iterative development will ensure the case remains a practical and high-yield resource for residents as they prepare for certification.

**Topics:**

Hyperthyroidism, communication, clinical decision-making case, certifying exam, thyroid storm, endocrinology.

## USER GUIDE

**Table:** 

List of Resources:	
Abstract	260
User Guide	263
For Examiner Only	267
Certifying Exam Assessment	274
Stimulus	276
Debriefing and Evaluation Pearls	287

**Learner Audience:**

All emergency medicine physicians in training.

**Time Required for Implementation:**

Case: Clinical Decision-Making cases are 15 minutes as directed by American Board of Emergency Medicine (ABEM).

Debriefing: 5 minutes

**Recommended number of learners per instructor:**

This case can be used to provide a mock certifying exam experience for emergency medicine residents, either in isolation or alongside additional cases to make the experience more realistic in preparation for the exam. For realism and accurate practice, we suggest arranging the case with a 1:1 ratio of faculty and learner. This case could be run with multiple learners for group practice. An additional use of the case would be to have a resident take the role of facilitator to gain an examiner’s perspective. The entire case, including feedback, should take 20 minutes, with 15 minutes for the case and 5 minutes for feedback.

**Topics:**

Hyperthyroidism, communication, clinical decision-making case, certifying exam, thyroid storm, endocrinology.

**Objectives:**

By the end of the session, learners will be able to:

1. Verbalize key pertinent historical and physical exam findings in a young female patient presenting with altered mental status.
2. Formulate a prioritized differential diagnosis based on the history and physical exam.
3. Order appropriate diagnostic studies and recognize abnormalities suggesting thyroid storm.
4. Describe pathophysiology, management, and rationale of sequential pharmacologic therapy in thyroid storm.
5. Communicate patient’s medical care and course to family.
6. Review essential disposition actions including consultations and level of care for admission.

### Linked objectives, methods and results

This clinical decision-making case centers on a 33-year-old female who presents to the emergency department with altered mental status. The facilitator begins by providing minimal initial information, prompting the learner to identify the focused historical elements they would obtain from the patient, EMS, family members, or bystanders, including information relevant to endocrine, infectious, or other causes of altered mental status (Objective 1). The learner is then asked to describe the components of a targeted multisystem physical exam and explain the relevance of specific findings, such as neurologic status, cardiovascular abnormalities, fever, skin changes, or signs suggestive of thyroid dysfunction or infection (Objective 1).

Next, the learner must formulate a prioritized differential diagnosis that includes both common and life-threatening etiologies of altered mental status in a young woman (Objective 2). Based on their assessment and differential, the learner will be required to select appropriate diagnostic studies and accurately interpret key clinical data, including laboratory values, urinalysis, ECG, imaging, and point-of-care tests (Objective 3). The diagnostic results in this case include leukocytosis, a nitrite-positive urinalysis, a normal glucose level, a normal head CT, and a suppressed thyroid-stimulating hormone (TSH) with elevated T3 and free T4. An ECG is provided that shows atrial fibrillation with rapid ventricular response, which the learner must recognize and incorporate into their diagnostic reasoning.

With these results, the learner is expected to initiate appropriate emergency department management for thyroid storm, including starting a beta-blocker, administering antithyroid medication, providing corticosteroids, offering supportive care, and considering empiric antibiotics when infection is suspected (Objective 4). The learner must then explain the rationale for the correct sequence of thyroid storm medications—beta-blocker followed by thionamide, then iodine, and corticosteroids—and identify relevant treatment endpoints such as heart rate, temperature, and mental status (Objective 4).

As the scenario progresses, the learner should reassess the patient and adapt the management plan based on evolving clinical information, changes in vital signs, and response to treatment (Objective 4). Throughout the case, the learner is expected to verbalize their medical decision- making, justify diagnostic and therapeutic actions, and communicate their interpretation of abnormal findings (Objective 4).

As the patient stabilizes, the facilitator introduces the patient’s husband, prompting the learner to provide anticipatory guidance, including a clear explanation of the diagnosis, treatment plan, expected hospital course, and next steps after admission (Objective 5). The learner must then identify appropriate consultants—including endocrinology and internal medicine—and determine that the patient requires ICU-level care based on her clinical status (Objective 6). Throughout the encounter and debrief, the learner is expected to demonstrate understanding of the pathophysiology of thyroid storm, including its systemic manifestations and common triggers, and connect this knowledge to the clinical presentation (Objective 4).

### Recommended pre-reading for instructor

- Farooqi S, Raj S, Koyfman A, Long B. High risk and low prevalence diseases: Thyroid storm. *Am J Emerg Med*. 2023;69:127–135. doi:10.1016/j.ajem.2023.03.035

### Results and tips for successful implementation

#### Tips for successful implementation

For additional realism, programs may incorporate a second faculty member to serve exclusively as the examiner, allowing the facilitator to focus on case delivery and prompts. The faculty facilitator followed a standardized script designed to replicate the new ABEM certifying exam format, ensuring consistency, realism, and educational relevance. During the case, residents were presented with a brief clinical stem and were required to verbalize both their patient assessment and their broader clinical reasoning. When available, a second faculty examiner used a structured checklist to capture communication behaviors and critical decision-making points.

#### Methods

The case was co-developed by three faculty members with expertise in emergency medicine and simulation-based education. It underwent external peer review to assess accuracy of the clinical content, clarity of instructions, and educational value. Reviewers used the Simulation Scenario Evaluation Tool (SSET) to provide structured feedback on case progression, realism, objectives, and alignment with assessment standards.

Following peer review, the case was piloted at two academic training sites and at the Society for Academic Emergency Medicine Annual Meeting. We employed an iterative trialing process with a convenience sample of EM residents. Residents and faculty participants completed usability surveys consisting of Likert-scale items (1 = strongly disagree to 5 = strongly agree) and open-ended feedback. Data were collected via QualtricsR (Provo, UT) and analyzed in ExcelR (Microsoft, Redmond, WA). The project was deemed exempt by the Boston University Institutional Review Board.

Examiner experience feedback survey:

1. What regional area do you practice in? Northeast, South, Midwest, West
2. What is your practice environment? Urban, Suburban, Rural
3. What is your role? Residency leadership, core faculty, non-core faculty, resident
4. Please rank your agreement with the following questions regarding the CURRICULUM guide:
  - I would like to use the curriculum for ABEM certifying exam practice: 1–5
  - I thought the curriculum was easy to use
  - I found the materials in this curriculum were well integrated
  - I would imagine that most people would learn to use this curriculum very quickly
  - I felt very confident using the curriculum for ABEM certifying exam practice
5. CURRICULUM General notes/comments
6. Please rank your agreement with the following questions regarding the CASE guide:
  - I would like to use this case for ABEM certifying exam practice: 1–5
  - I thought the case was easy to use
  - I found the materials in this case were well integrated
  - I would imagine that most people would learn to use this case very quickly
  - I felt very confident using the case for ABEM certifying exam practice
7. CASE General notes/comments
8. Are there any clinical topics you would like to see in this case format?

Examinee experience feedback survey:

1. Where are you testing this case? BMC, RWJ, SAEM, Northwell, Other___
2. What regional area do you practice in? Northeast, South, Midwest, West
3. What is your practice environment? Urban, Suburban, Rural
4. What is your experience level? PGY1, PGY2, PGY3, PGY4, Fellow, Faculty
5. Please rank your agreement with the following questions:
  - The written materials were clear
  - The verbal instructions were clear
  - This was helpful practice for the ABEM certifying exam
6. What would you change?
7. What was helpful?
8. Are there any clinical topics you would like to see in this case format?
9. Any other feedback/comments?

#### Iterative Piloting

- Round 1: Two EM residents at an academic site tested the case with faculty evaluation using the SSET and resident usability surveys.
- Round 2: At the SAEM Annual Meeting (May 2025, Philadelphia, PA), two facilitators and three residents participated, again using the SSET and usability surveys.
- Round 3: At a second academic site, seven PGY-3 residents completed the case and provided structured feedback.

Across all sites, revisions were made iteratively to the case content, facilitator script, and assessment tools, resulting in improved clarity, usability, and educational impact.

#### Results

The SSET evaluations were strongly positive (Table 1). Facilitators (n = 3) consistently rated the case objectives, critical actions, and supporting materials as clear. They agreed or strongly agreed that the case was appropriate for the learners’ knowledge and skill level, that the critical actions aligned with the stated objectives, and that the clinical course adhered to the ABEM format. Mean ratings ranged from 4 to 5 for ease of use, and facilitators indicated that their colleagues would also find the materials accessible. The debriefing plan component of the SSET received the lowest rating, with a mean Likert score of 1, reflecting that the initial manuscript did not present a sufficiently detailed debriefing strategy. Based on this feedback, the case was revised to include a more structured and robust debriefing plan, which improved the overall clarity and educational utility of the materials. Overall, they described the case as well-integrated, expressed confidence in facilitating it, and endorsed its value for ABEM certifying exam preparation.

Given the very small number of respondents, these early findings should be interpreted cautiously. Only one resident completed the post-session survey during the pilot. This PGY-2 resident rated the written materials as very clear (5/5), the verbal instructions as clear (4/5), and the overall helpfulness of the case for ABEM exam preparation as 5/5. In open-ended feedback, the learner highlighted the value of “step-by-step instructor guidance,” and recommended minor clarification regarding expectations for the history and physical examination. The resident also identified “toxidromes” as a topic of interest for future cases developed in this format.

Faculty feedback, although also limited (n = 2), demonstrated similarly positive impressions. Both facilitators indicated they would use the case for ABEM preparation (ratings 4 and 5). Ratings reflected that the case was generally easy to use (3 and 5), well-integrated (4 and 5), and easy to learn (4 and 5). Confidence in facilitating the case ranged from 3 to 5. Across comments, facilitators endorsed the clarity of the materials, the organization of the case, and its utility as a certifying exam board practice tool.

Taken together, these preliminary results suggest high acceptability and perceived usefulness among early users, but further evaluation with a larger and more diverse group of residents and faculty will be necessary to draw more definitive conclusions.

## Figures and Tables

**Table:** 

**I. History**		Yes	No
**1a**	Current symptoms that prompted Emergency Department visit		
**1b**	Symptom duration		
**1c**	Triggers for Thyroid Storm (infection, trauma, surgery, medications, recent medication changes, etc.)		
**1d**	Medications		
**1e**	Past Medical History		
**II. Physical Examination**			
**2a**	HEENT Exam (pupillary exam, mucous membranes, proptosis, lid lag)		
**2b**	Neck Exam (goiter or meningismus)		
**2c**	Cardiac Exam (irregularly irregular rhythm, rate)		
**2d**	Neuro Exam (evaluation of altered mental status looking for patient orientation, focal neurologic deficits)		
**III. Differential Diagnosis**			
**3a**	Thyroid Storm		
**3b**	2^nd^ appropriate diagnosis (see Prompt 3 scoring guidelines above)		
**3c**	3^rd^ appropriate diagnosis (see Prompt 3 scoring guidelines above)		
**IV. Diagnostic Studies**			
**4a**	TSH/Free T4/T3		
**4b**	CBC		
**4c**	Drug/Toxicology Screen		
**4d**	Pregnancy Test		
**4e**	ECG		
**4f**	Urinalysis and Urine culture		
**4g**	Consider Sepsis: Blood culture and lactic acid		
**V. Broad Concepts: Head CT**			
**5**	Able to provide three findings on a head CT that would cause altered mental status		
**VI. Broad Concepts: ECG**			
**6**	Correct interpretation of EKG (Atrial fibrillation with RVR)		
**7**	Able to provide rationale for indications of rate control in a patient in Afib with RVR		
**VII. Treatment and Other Actions**			
**8a**	Beta Blocker 1st		
**8b**	Propylthiouracil/methimazole 2nd		
**8c**	Steroid 3rd or 4th (can be given before or after iodine)		
**8d**	Iodine 3rd or 4th (should be given 1 hour after PTU or methimazole)		
**8e**	IV antibiotics for UTI (after urine culture is obtained)		
**VIII. Final Diagnosis**			
**9**	Urinary Tract Infection		
**10**	Thyroid Storm		
**IX. Communication/Disposition/Transition of Care**			
**11a**	Informed conversation with the husband about diagnosis, management, and next steps in layman’s terms		
**11b**	Consultation to Endocrine		
**11c**	Admission to ICU		

**Table:** 

**Candidate Task Sheet**	
**#1**	**Emergency Department Admitting Form**
**#2**	**Historical Information**
**#3**	**Physical Exam Findings**
**#4**	**ECG**
**#5**	**Laboratory and Point-of-Care Studies**
**#6**	**Chest X-ray**
**#7**	**Head CT**

**Table:** Clinical Decision-Making Task Sheet

**CASE PARAMETERS**
This is a 15-minute case You will interact with two examiners. This is an interview style without role playing; you should simply reply to the questions asked. You may be interrupted to move you through the case; this is not a reflection of your performance.
**PATIENT INFORMATION**
33-year-old female presents to the ED via EMS for altered mental status.
**VITAL SIGNS**
BP: 158/99 P: 142 R: 124 T: 40.1° C 02Sat: 98%
**TASK STATEMENT**
Your tasks are as follows: List pertinent elements of a focused history and physical exam Develop an appropriate differential and/or provisional diagnosis Select and interpret appropriate studies Articulate appropriate patient management including discharge instructions

**STIMULUS 1:** Emergency Department Admitting Form

**Patient Information**	
**Patient Name**	**Jessica** Gonzalez
**Age**	33 yo
**Gender**	F
**Method of Arrival**	EMS
**General Appearance/History of Present Illness**	She is diaphoretic, confused and can only provide her name.
**Vital Signs on ED Arrival**	BP: 158/99 P: 142 R: 24 T: 40.1° C O2 sat:98%

**STIMULUS 2 t7-jetem-10-5-ce260:** Historical Information

**History of Present Illness/Description of Event**	
The patient is unable to give any history due to confusion, but the husband gives the history at bedside. The patient has been complaining of some burning with urination for the past three days. She has been taking over the counter cranberry supplement for these symptoms. The patient’s husband went home to check on his wife after her coworkers called him stating that she did not show up to work that morning. He found her diaphoretic, laying on the couch. She was confused and could only tell her husband her name. He called EMS, who transported the patient to the emergency department for evaluation and treatment.	
**History**	
**Past Medical History**	Celiac Disease
**Past Surgical History**	None
**Medications**	Multivitamin, Oral Contraception
**Allergies**	No known drug allergies
**Social**	None

**STIMULUS 3 t8-jetem-10-5-ce260:** Physical Exam Findings

**Physical Examination**	
**General**	Laying on the stretcher, diaphoretic.
**HEENT**	Atraumatic, normocephalic. Pupils are 3 mm bilaterally and reactive to light. Normal Conjunctiva. Proptosis bilaterally and lid lag with extraocular movements. Normal tympanic membranes bilaterally and external ears normal. Dry mucous membranes; no oropharyngeal edema, erythema, or exudates.
**Neck**	Supple, full range of motion. No meningismus. Palpable goiter.
**Cardiovascular**	Irregularly irregular rhythm, rapid rate, no murmurs.
**Respiratory**	Tachypneic. Breath sounds clear and equal bilaterally without any wheezing, crackles, or ronchi.
**Abdomen**	Soft, non-distended. Suprapubic tenderness to palpation. No rebound tenderness or involuntary guarding.
**Genital**	No vaginal discharge or bleeding. Normal, closed cervix. No cervical motion tenderness. No adnexal tenderness or masses.
**Back**	No midline tenderness to palpation. Right costovertebral angle tenderness.
**Extremities**	Normal range of motion of joints with no rigidity.
**Neuro**	Agitated. Oriented to self only. Opens eyes to voice. Follows commands in all four extremities. Cranial nerves II–XII intact. 5/5 strength and intact sensation to light touch in all four extremities. No clonus. Hyperreflexia.
**Skin**	Diaphoretic. No rashes.
**Lymph**	No lymphadenopathy.
**Psych**	Unable to completely assess since patient is only able to state her name.

**STIMULUS 4:**
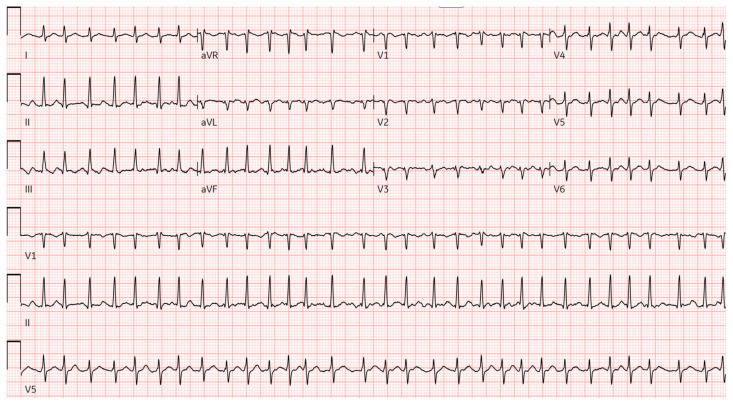
ECG Image Source: Authors’ own image.

**STIMULUS 4 t9-jetem-10-5-ce260:** Laboratory and Point-of-Care Studies

Lab Panel	Type	Units	Normal Values
Point-of-Care	Glucose	90 mg/dL	70–105 mg/dL
	HCG	Negative	Negative
Complete Blood Count	WBC	16.1 × 10^3^/μL	4–11 × 10^3^/μL
	HCT	38.80%	36–47% female
			41–51% male
	HGB	12.9 g/dl	12–14 g/dL female
			14–17 g/dL male
	PLT	270 × 10^3^/μL	150–450 × 10^3^/μL
	Neutrophils	66% (10,626)	
	Lymphocytes	26% (4186)	
	Monocytes	5% (402)	
	Eosinophils	2.5% (402)	
	Basophils	0.5% (81)	
Basic Metabolic Panel	Na	142 mEq/L	136–145 mEq/L
	K	4.4 mEq/L	3.5–5 mEq/L
	Cl	107 mEq/L	98–106 mEq/L
	CO_2_	24 mEq/L	23–28 mEq/L
	BUN	13 mEq/dL	8–20 mEq/dL
	Creatinine	0.68 mg/dL	0.7–1.3 mg/dL
	Glucose	95 mg/dL	70–105 mg/dL
	Calcium	9.4 mg/dl	9.0–10.5 mg/dL
Liver Function Panel	AST	15 U/L	0–35 U/L
	ALT	11 U/L	0–35 U/L
	Alk Phos	49 U/L	36–92 U/L
	T bili	0.4 mg/dL	0.3–1.2 mg/dL
	D bili	0.2 mg/dL	0.0–0.3 mg/dL
	Albumin	4.1 g/dL	3.5–5.0 mg/dL
	Total Protein	7.3 g/dL	6–8.3 mg/dL
Urinalysis	Specific Gravity	1.023	1.005–1.030
	pH	5.5	4.5–8.0
	Protein	30 mg/dL	<150 md/dL
	Glucose	Negative	Negative
	Ketones	Negative	Negative
	Bili	Negative	Negative
	Blood	Small	None
	LE	Large	None
	Nitrite	Positive	Negative
	Color	Yellow	Yellow
	WBC	1123/hpf	<2–5 WBC/hpf
	RBC	35 hpf	<2 RBC/hpf
	Bacteria	3+	None
Arterial Blood Gas	pH	7.41	7.37–7.44
	PO_2_	94 mm Hg	80–100 mm Hg
	PCO_2_	37 mm Hg	35–45 mm Hg
	HCO_3_	26 mmol/L	22–26 mmol/L
	Lactate	2.4 mEq/L	0.4–2.3 mEq/L
Thyroid Function Studies	TSH	0.04 mIU/L	0.350 – 3.600 mIU/L
	Free T4	17.4 ng/dl	0.70 – 1.37 ng/dL
	T3	308 ng/dL	80 to 200 ng/dL
Troponin	Troponin	<0.01 ng/ml	<0.01 ng/ml
Serum Tox Screen	Acetaminophen	Negative	Negative
	Ethanol	Negative	Negative
	Salicylate	Negative	Negative
Urine Tox Screen	Acetaminophen	Negative	Negative
	Amphetamine	Negative	Negative
	Barbiturate	Negative	Negative
	Benzodiazepine	Negative	Negative
	Buprenorphine	Negative	Negative
	Cannabinoid	Negative	Negative
	Cocaine	Negative	Negative
	Ethanol	Negative	Negative
	Methadone	Negative	Negative
	Opiate	Negative	Negative
	Oxycodone	Negative	Negative
	Salicylate	Negative	Negative
	Tricyclic	Negative	Negative

**STIMULUS 6 f2-jetem-10-5-ce260:**
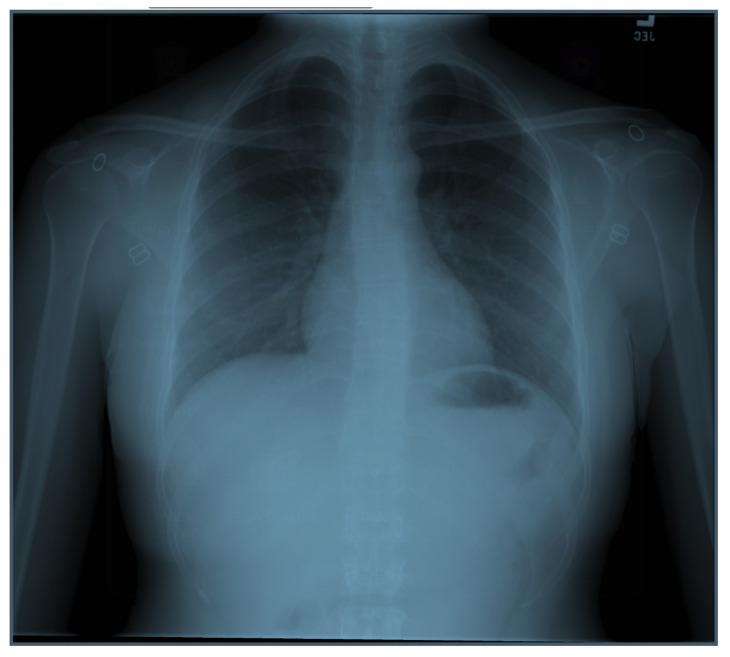
Chest X-Ray Image Source: Authors’ own image.

**STIMULUS 7 f3-jetem-10-5-ce260:**
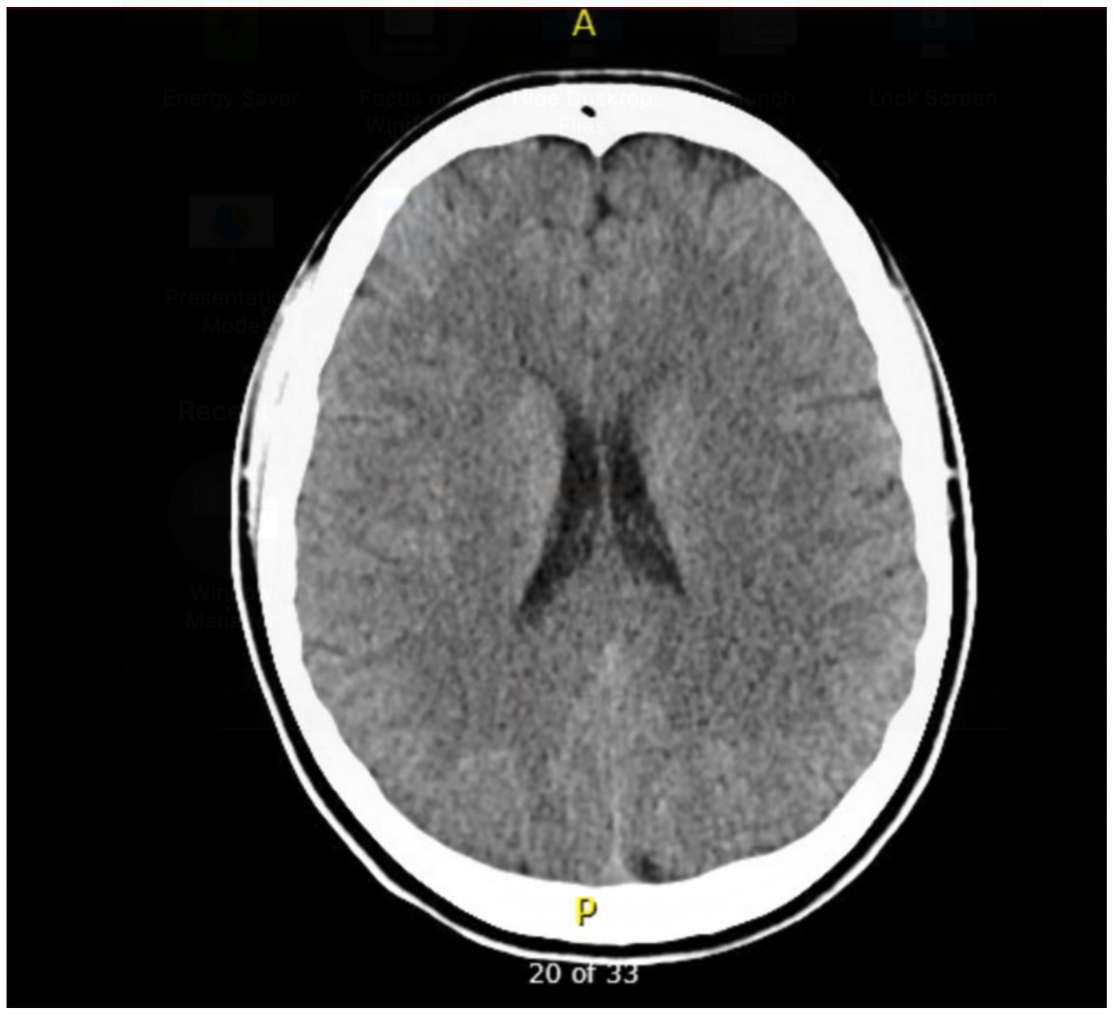
Head CT Image Source: Authors’ own image.

**Table 1:** SSET Evaluation Raw Results (3 peer reviewer respondents)

Learning Objectives			
Objectives pertain to the skill level and knowledge base of the target audience	5	5	5
Objectives are specific	5	5	5
Objectives are measurable	5	5	5
Objectives are action-oriented	5	5	5
Objectives are relevant	5	5	5
Objectives are time-specific	5	5	5
Objectives specify the types of knowledge/skills expected to be gained by completing the simulation exercise	5	5	5
Clinical Context/Scenario Overview			
Clinical context elements pertain to the skill level and knowledge base of the target audience	5	5	5
Clinical context elements support the learning objectives of the simulation-based experience	5	5	5
Critical Actions			
Attainable in accordance with the skill level and knowledge base of the target audience	5	5	5
Support the learning objectives	4	5	5
Directly observable	3	5	5
Patient States			
Clinically appropriate for case progression	5	5	4
Accounts for multiple management pathways a learner may take	4	5	4
Effective for meeting the learning objectives	5	5	4
Effective for eliciting the outline critical actions	5	5	4
Scenario Materials and Resources			
Provides a list of necessary materials, equipment and human resources to support facilitation	5	5	5
Provides a list of necessary patient historical data, atrioventricular (AV) stimuli and clinical resources to support case facilitation	5	5	5
Debriefing Plan			
Presents a clear debriefing plan	1	1	1
Provides supporting materials with References to aid debrief	4	5	1
Does the case follow the format provided by the ABEM case example?	5	5	5
Does the case follow the format provided by the JETem template?	4	5	5
**Global Rating Scale** What is the overall rating of the sim case quality?	80	91	93

## FOR EXAMINER ONLY

### Clinical Decision-Making Case: Thyroid Storm Summary

**Diagnosis:** Thyroid storm

**Case Summary:** This clinical decision-making case involves a 33-year-old woman who presents to the emergency department with altered mental status. The facilitator guides the learner through a series of prompts to elicit their clinical reasoning, beginning with the need to gather a focused history from available sources. The patient is unable to provide her own history, but her husband reports that the patient has had several days of burning with urination and has been using cranberry supplements. He found her confused and diaphoretic at home. The physical exam reveals signs concerning thyrotoxicosis, including bilateral eyelid proptosis with lid lag, a palpable goiter, agitation, hyperreflexia, and an irregularly irregular tachycardia. As the case unfolds, the learner is expected to develop a differential diagnosis for altered mental status, order appropriate diagnostic studies, and interpret the results. Key findings include suppressed thyroid stimulating hormone (TSH) with elevated triiodothyronine (T3) and free thyroxine (T4), atrial fibrillation with rapid ventricular response (RVR), and evidence of a urinary tract infection. The learner must then make a diagnosis of thyroid storm and initiate appropriate treatment for thyroid storm, including beta-blockers, antithyroid medications, corticosteroids, and iodine in the correct sequence. Throughout the scenario, the learner is asked to explain their clinical decisions, describe endpoints of therapy, reassess the patient’s condition, and communicate the diagnosis and treatment plan to the patient’s family. The case concludes with decisions regarding consultation and intensive care unit (ICU) admission.

### Clinical Decision-Making Case: Thyroid Storm Examiner Script

**Case Introduction:**

“Hello Doctor, this is a clinical decision-making case. There is no role playing. In response to the questions I will ask, please give me a LIST of information you would gather to come to a final diagnosis. At times, I may interrupt you to move you through the case; this is not a reflection of your performance. You will have 15 minutes to complete the case. Before we begin, do you have any questions?”

“All right, our time begins now. The patient we will be discussing is a 33-year-old female who presents to the emergency department with altered mental status. Her initial information is here on the screen.”

Provide Learner Stimulus #1:

#### HISTORY

Prompt 1:

“After you have reviewed that information, what specifically would you ask of the patient, her family, or bystanders?”

Scoring Guidelines:

Rationale: Learners should obtain history from the patient’s husband given that she is unable to provide history. They should ask the reason the patient was brought to the Emergency Department, current symptoms, onset of symptoms, potential triggers for thyrotoxicosis (infection, surgery, trauma, medications the patient is currently on or any recent medication changes), patient’s past medical history and current medications as well as social history including alcohol and drug use.

#### PHYSICAL EXAMINATION

Prompt 2:

“Thank you, doctor. Now please discuss specifically what you would be assessing on the physical exam of the patient.”

Facilitator (follow-up prompts):

- “Can you clarify what you are looking for on your cardiac exam?”
- “Can you clarify what you are looking for on your neurologic exam?”
- “Can you clarify what you are looking for on your HEENT exam?”
- “Can you clarify what you are looking for on your neck exam?”

Scoring Guidelines:

Rationale: Learners should examine the patient with a broad differential given the medical history and vital signs:

The patient’s cardiac exam should include evaluating the patient’s rate and rhythm.

The neurologic exam should evaluate for possible causes of altered mental status, evaluating the patient’s mentation, orientation, and looking for focal neurologic deficits.

The HEENT exam should evaluate the pupils to make sure they are equal and reactive, evaluate mucous membranes to look for signs of dehydration, look for evidence of proptosis or lid lag, and their neck exam should evaluate for meningismus as well as a goiter to rule out differentials of meningitis or thyrotoxicosis.

“Thank you, doctor. Here are the additional historical and physical exam findings. They are also displayed on the screen.”

Provide Learner Stimuli #2 and 3 and read pertinent information.

“The patient is unable to give any history due to confusion, but her husband provides history at bedside. She has had burning with urination for the past three days and was taking OTC cranberry supplements. He found her diaphoretic and confused on the couch after she didn’t show up for work.

Past Medical History: Celiac disease. Medications: Daily multivitamin, oral contraception.”

“On exam, the patient is diaphoretic. There is no head trauma. The pupils are 3 mm and reactive. She has bilateral proptosis with lid lag. There is no meningismus. She has a palpable goiter. She has irregularly irregular tachycardia. She is tachypneic with clear lungs. She has suprapubic tenderness and right-sided right costovertebral angle (CVA) tenderness. She is agitated and oriented only to self. Cranial nerves are intact. She has hyperreflexia, no clonus.”

#### DIFFERENTIAL DIAGNOSIS

Prompt 3:

“At this point, based on what you now know, what are the top three items in your differential diagnoses for this patient at this time?”

Scoring Guidelines:

Rationale: Examples of appropriate differentials include: thyroid storm/thyrotoxicosis, sepsis secondary to UTI, sepsis secondary to meningitis/encephalitis, serotonin syndrome, neuroleptic malignant syndrome, sympathomimetic toxicity, anticholinergic toxicity, alcohol withdrawal, salicylate toxicity, heat stroke, pheochromocytoma, or malignant hyperthermia.

#### DIAGNOSTIC STUDIES

Prompt 4:

“Based on your working differential, what diagnostic studies, if any, would you order or perform at this time?”

Scoring Guidelines:

Rationale: Diagnostic studies evaluating for the above differential diagnoses. Labs that should not be missed on this patient include a point-of-care blood glucose, CBC, BMP, LFTs, urinalysis, thyroid function studies, troponin, serum toxicology screen (acetaminophen, ethanol, salicylate), and urine toxicology screen. Other diagnostic studies that should be performed include an EKG, chest x-ray, and head CT.

Prompt 5:

“Not in regard to this specific patient, what would you be looking for if obtaining a head CT in a patient with altered mental status? Please give three diagnoses/differentials.”

Scoring Guidelines:

Rationale: Learners should be able to give at least three differentials or diagnoses that they would be looking for when ordering a CT on a patient with altered mental status. Appropriate answers include intracranial hemorrhage, ischemic stroke/large vessel occlusion, space occupying lesion/mass/tumor, signs of increased intracranial pressure or herniation, cerebral edema, infection (meningitis/encephalitis, abscess), trauma, hydrocephalus, Posterior Reversible Encephalopathy Syndrome (PRES).

Prompt 6:

“An electrocardiogram has been performed. Please interpret it.” PROVIDE STIMULUS 4.

Scoring Guidelines:

Rationale: Learners should be able to accurately interpret the EKG stimulus which shows atrial fibrillation with rapid ventricular response.

Prompt 7:

“Are there any immediate interventions you would do based on the ECG above?”

Scoring Guidelines:

Rationale: Learner may decide to give the patient IV fluids as the first intervention, or they may decide to rate control using diltiazem or metoprolol.

#### TREATMENT AND OTHER ACTIONS

Prompt 8:

“The patient appears to be in atrial fibrillation with RVR. How do you decide when to rate control a patient with this finding?”

Scoring Guidelines:

Rationale: This question is to make sure the learner understands indications for rate control in a patient in atrial fibrillation with rapid ventricular response. First, if the patient is unstable (systolic blood pressure < 90 mmHg or mean arterial pressure < 65, evidence of shock or poor perfusion, myocardial ischemia, severe dyspnea), they should not be rate controlled with a beta-blocker or calcium channel blocker; synchronized cardioversion may be necessary. If the patient is stable, the learner should try to determine the underlying cause of the patient’s tachydysrhythmia. If the tachydysrhythmia is due to a likely physiologic or compensatory response (fever, sepsis, pulmonary embolism, hyperthyroidism), the patient should not be rate controlled until the underlying cause is addressed. If the patient is hemodynamically stable and there is no acute reversible cause or evidence that the tachydysrhythmia is compensating for another problem, the patient can safely be rate controlled.

“Thank you, doctor. Here are the results of the diagnostic studies you ordered.”

Provide Learner Stimuli #5, #6, #7

Prompt 9:

“At this point, what treatments or actions would you take in the emergency department for this patient?”

Facilitator (as needed):

- “Could you elaborate more, please?”
- “Which medications would you give for her underlying medical condition, and in what order?”
- “Can you explain why the order is important?”
- “What endpoints are you looking for to determine patient improvement?”

Scoring Guidelines:

Rationale: By this point in the case, learners should recognize decreased TSH as well as elevated T3 and Free T4 as well as a urinary tract infection (UTI) as a potential trigger for thyroid storm. Learners should obtain a urine culture and then give antibiotics for the UTI. To treat thyroid storm, learners should first give a beta blocker such as propranolol; next, they should give propylthiouracil (PTU) or methimazole; one-hour after PTU or methimazole, iodine should be given, and a steroid should also be given but can be given before or after the iodine.

#### COMMUNICATION WITH FAMILY

“Thank you, doctor. The actions you described are now being performed.

Based on everything that has happened in the case so far, the husband would like an update.”

Prompt 10:

“Based on everything you know now, what is the final diagnosis?”

Scoring Guidelines:

Rationale: Urinary tract infection leading to thyroid storm.

Prompt 11:

“What would you discuss with the husband about your evaluation, treatment, and further management? What would the next steps be?”

Scoring Guidelines:

Rationale: Verbalizing a diagnosis in plain language for the patient/family member, next steps, disposition, and return precautions. The learners should inform the husband about the diagnosis; they should tell him that based on the labs and imaging it appears his wife had a urinary infection that then unmasked a thyroid problem the patient likely already had. They should tell him that the patient will be treated with antibiotics for the urinary infection as well as multiple other medications to control the patient’s thyroid. They should inform him that the patient will be admitted to the intensive care unit (ICU) while these medications are given so the patient’s vital signs and labs can be closely monitored during treatment. They should also inform him that upon discharge from the hospital, the patient will be on thyroid medications and need to follow-up with an endocrinologist.

#### DISPOSITION/TRANSITION OF CARE

Prompt 12:

“Okay, doctor. (If not already stated) Would you like to consult with anyone on this patient? Where would you like to admit this patient to?”

Scoring Guidelines:

Rationale: Learner should verbalize that endocrinology needs to be consulted, and the patient needs to be admitted to an ICU level of care.

*Thank you, Doctor. That concludes this case.*

*Please tear up your notes.*

## CERTIFYING EXAM ASSESSMENT

*Clinical Decision-Making Case: Thyroid Storm*

Learner: _________________________________________

**Summative and formative comments:**

### Stimulus Inventory

[Table t7-jetem-10-5-ce260] [Table t8-jetem-10-5-ce260] [Table t9-jetem-10-5-ce260]

[Fig f2-jetem-10-5-ce260] [Fig f3-jetem-10-5-ce260]

## DEBRIEFING AND EVALUATION PEARLS

### Clinical Decision-Making Case: Thyroid Storm

#### Focused History and Targeted Multisystem Physical Exam

During debriefing, emphasize that focused history-taking is essential because patients in thyroid storm often present with altered mental status and cannot provide reliable information. Key elements include symptom onset, fever, agitation, medication adherence, recent illness or infection, trauma, surgery, iodinated contrast exposure, and medications such as amiodarone. Infection and medication nonadherence are the most common precipitants, although no trigger is identified in up to 43% of cases.^1^,^2^ Learners should recognize that thyroid storm occurs most frequently in women, often between ages 20–50 and may be associated with underlying Graves disease.^1^ Presentations are often nonspecific: anxiety, palpitations, gastrointestinal symptoms, and altered mental status are common early manifestations.^2^

Debrief whether learners appropriately obtained collateral information from EMS, family, or bystanders and how this influenced their diagnostic reasoning.

A comprehensive physical exam is crucial because thyroid storm affects multiple organ systems simultaneously. Typical findings include hyperpyrexia, tachycardia, atrial fibrillation, vomiting/diarrhea, hepatic dysfunction, and central nervous system (CNS disturbance.^1^,^2^ Learners should connect findings such as agitation, tremor, high-output heart failure, or diaphoresis to thyrotoxicosis. A classic clinical triad—tachycardia, hypertension, and altered mental status—should raise suspicion. Signs of high-output heart failure (jugular vein distention, edema) may appear later but are important to monitor. Discuss the significance of palpable goiter or exophthalmos (present in ~20% of cases) while reminding learners that deep palpation of the thyroid is discouraged due to rare reports of precipitation of thyroid storm.^2^

#### Prioritized Differential Diagnosis

Debrief how learners differentiated thyroid storm from other life-threatening causes of altered mental status. The differential should include sepsis/septic shock, CNS infection, diabetic ketoacidosis, adrenal crisis, toxicologic syndromes (serotonin syndrome, sympathomimetic toxicity), neuroleptic malignant syndrome, intracranial hemorrhage, pheochromocytoma, heart failure, and tachyarrhythmias. Emphasize that thyroid storm has many mimics, and early diagnostic confusion is common. Learners should understand that the diagnostic approach must reflect the differential—ordering TSH and free T4/T3 alongside sepsis, metabolic, and cardiac evaluations is critical. In most thyroid storm cases, TSH is low and free T4/T3 are elevated. Explore how historical features, vital signs, and initial testing help learners refine their differential.

#### Diagnostics and Data Interpretation

Thyroid storm is primarily a clinical diagnosis, but laboratory and imaging data support the assessment. Both references recommend obtaining CBC, electrolytes, renal and liver studies, TSH, free T4/T3, ECG, chest imaging, urinalysis, and additional testing based on presentation.^1^,^2^

Learners should identify key abnormalities such as suppressed TSH, elevated free T4, leukocytosis, hyperglycemia, and liver function test derangements. Atrial fibrillation is seen in 10–35% of thyroid storm patients and should be recognized as a high-risk finding.^2^ Debrief how learners integrated these findings and whether they accurately interpreted at least five data points to support or refute thyroid storm.

#### Pathophysiology

Debrief by connecting pathophysiologic mechanisms to the patient’s clinical presentation. Thyroid storm reflects excessive thyroid hormone action, leading to increased adrenergic receptor density, heightened catecholamine responsiveness, rapid T4→T3 conversion, and loss of homeostatic control between thyroid hormone and the sympathoadrenal system. 1, 2 These mechanisms explain symptoms such as tachycardia, agitation, hyperthermia, gastrointestinal upset, and multisystem decompensation. Understanding these processes reinforces why treatment must be sequenced and aggressive.

#### Management and Rationale of Pharmacologic Therapy

Debrief the learner’s approach to early management, which must be rapid and simultaneous. Appropriate interventions include administering beta-blockers, thionamides, corticosteroids, IV fluids (if dehydrated), cooling measures, benzodiazepines for agitation, and empiric antibiotics when infection is suspected.^1^, ^2^ Discuss potential pitfalls such as giving beta-blockers to a patient with severe heart failure or delaying fluids in dehydrated patients. Reinforce that early resuscitation, stabilization of the cardiovascular system, and management of hyperthermia and agitation are essential components of care. The correct pharmacologic sequence—beta-blocker → thionamide → iodine → corticosteroids—is critical because administering iodine before thionamides can increase thyroid hormone synthesis. 1, 2

Debrief each medication’s role:

- Beta-blockers: Reduces adrenergic hyperactivity; propranolol partially reduces peripheral T4→T3 conversion.
- Thionamides: Propylthiouracil **(**PTU) preferred initially): Inhibits hormone synthesis; PTU also blocks peripheral T4→T3 conversion.
- Iodine (given 1 hour later): Blocks release of preformed hormone.
- Steroids: Reduces peripheral conversion and helps treat possible adrenal insufficiency. Steroids can be given before or after iodine.

Discuss expected endpoints of improvement: lowering heart rate, decreasing temperature, improving mental status, and stabilizing metabolic abnormalities.

Reassessment must occur frequently because thyroid storm patients can deteriorate rapidly.

Debrief how often learners reassessed vital signs, mental status, temperature, cardiac function, and overall clinical trajectory. The literature stresses the importance of monitoring for shock, arrhythmias, and evolving organ dysfunction. 1, 2

Discuss whether learners modified treatment appropriately by initiating vasopressors, adjusting fluids, escalating cooling, or addressing new arrhythmias.

#### Communication of Medical Decision-Making

High-acuity endocrine emergencies require transparent communication. Debrief how clearly the learner described their diagnostic reasoning, interpretation of abnormal findings, and prioritization of interventions. Discuss their use of closed-loop communication, team updates, and explanation of clinical uncertainty. Evaluate how well they maintained situational awareness during rapid patient changes.

#### Communication with Family

Learners should be able to explain thyroid storm to caregivers in clear, accessible terms—describing the diagnosis, rationale for interventions, anticipated ICU admission, potential complications, and overall hospitalization plan. Both sources emphasize that thyroid storm carries substantial morbidity and often requires aggressive ICU-level monitoring and treatment.^1^,^2^

Debrief how well learners balanced clarity, accuracy, and empathy.

#### Consultation and Disposition

Debrief whether learners correctly identified the need to involve endocrinology, internal medicine, critical care, and cardiology when appropriate. Thyroid storm universally requires ICU admission due to risks of cardiac failure, respiratory compromise, neurologic deterioration, and multisystem collapse. 1, 2

Discuss the clinical criteria learners used—such as severe tachyarrhythmia, heart failure, hypotension, or worsening mental status—to justify ICU disposition.

##### PEARLS Framework

- Reactions Phase
  *Goal: Allow the learner to process emotional and cognitive responses.*
  - “How did that scenario feel for you overall?”
  - “Was there a moment when things ‘clicked’ or the diagnosis became clear for you?”
  - “Was there a moment that felt particularly challenging or rewarding?”
- Description Phase
  *Goal: Establish a shared understanding of what happened.*
  - “Can you please share with me the summary of the case?”
- Analysis Phase
  *Goal: Facilitate reflection and provide structured feedback based on rubric scores.*
  - “What led you to consider thyroid storm as the diagnosis?”
  - “What were some alternative diagnoses you considered, and how did you rule them out?”
  - “Were there any early signs you may have missed that, in hindsight, pointed toward thyroid storm?”
  - “Did you feel confident in managing the sequence of therapies specific to thyroid storm?”
  - “How did you prioritize interventions — for example, rate control, supportive care, antithyroid therapies, or consults?”
  - “Was there a moment in the case where you weren’t sure what to do? What do you wish you had known then?”
- Application/Summary Phase
  *Goal: help learners consolidate key takeaways from the simulation and connect them to future clinical practice.*
  - “Was there a moment in the case where you weren’t sure what to do? What do you wish you had known then?”
  - “What were your biggest learning points from this simulation?”
  - “How will this case influence your approach to undifferentiated tachycardia or altered mental status in the ED?”
  - “What’s one specific skill or knowledge area you’d like to improve based on this case?”
